# How global is our research on child and adolescent mental health? A worldwide concentration and imbalance – and an opportunity for *JCPP* to take the lead in disseminating globally diverse research

**DOI:** 10.1111/jcpp.70163

**Published:** 2026-04-27

**Authors:** Sylia Wilson, Krupa Patel

**Affiliations:** ^1^ Institute of Child Development University of Minnesota Minneapolis MN USA; ^2^ Department of Psychology University of Minnesota Minneapolis MN USA

**Keywords:** Mental health

## Abstract

Mental health conditions, including among children and adolescents, are prevalent in the general population worldwide. Yet, much of our child and adolescent mental health research is not globally representative, with the vast majority of research conducted in samples in, and by researchers from, the United Kingdom and Western Europe, United States, Canada, Australia, and New Zealand. In this editorial, we highlight the *Journal of Child Psychology and Psychiatry* as well‐positioned to support and promote research on child and adolescent mental health conducted in globally diverse samples, by researchers from higher‐, middle‐, and lower‐income countries that is representative of and generalizable to children and adolescents throughout the world.

Readers of the *Journal of Child Psychology and Psychiatry* understand that child and adolescent health and well‐being is of critical importance. Concerns about child and adolescent mental health have been increasingly raised in recent decades, in conjunction with the growing prevalence worldwide of various psychiatric conditions (e.g., depression, anxiety, substance use, attention‐deficit/hyperactivity, and autism; Polanczyk, Salum, Sugaya, Caye, & Rohde, 2015). *JCPP* is widely recognized as the leading international journal of child and adolescent mental health – and it is well‐positioned to support and promote child and adolescent mental health research representative of the global population and conducted by researchers from around the world.

The need for globally representative research has long been recognized by *JCPP*. Almost 15 years ago, its editor at the time noted in the Journal's 9‐point mission statement the goal to ‘be truly international in orientation and values, content and the make‐up of contributors and readership’ (Sonuga‐Barke, 2012, p. 916). At the same time, challenges to conducting and publishing global research were also noted, including limitations in research resources, technical expertise, and scientific ‘knowhow’ (Sonuga‐Barke, 2014, p. 302). These and other challenges have resulted in a body of published research that lags behind laudable efforts to represent children and adolescents, and researchers, worldwide.

To quantify the countries in which research reported in *JCPP* has been conducted, as well as the countries of its study authors, we reviewed empirical studies published in *JCPP* over the past three decades.^1^ ^,^ ^2^ (See Figure 1 for an illustration of the major findings of the review and Table S1 for an overview of results.) Overall, in the 1,375 articles included in our review, reporting on 1,601 independent samples, research was conducted in 76 countries from around the world. However, and not surprisingly, though still sobering, almost all (95%) of the research was conducted in a much smaller subset of countries – the United States, United Kingdom and Western Europe, Canada, Australia, or New Zealand, with almost half of the samples from the United Kingdom or the United States – and there was no research conducted at all in most of the countries in Africa, Asia, or South America. These rates have held steady over the past 3 decades: 95% of studies published in the 1990s, 96% published in the 2000s, and 93% published in the 2010s were conducted in the United States, United Kingdom and Western Europe, Canada, Australia, and New Zealand. Moreover, although study authors represented 70 countries around the world, very few were from Africa, Asia, or South America (<3% collectively across these continents), and studies were almost exclusively first‐ (94%) or last/senior‐ (94%) authored by researchers from the United States, United Kingdom and Western Europe, Canada, Australia, or New Zealand. Notably, more than half (57%) of the studies that were conducted in countries other than in the United Kingdom and Western Europe, United States, Canada, Australia, and New Zealand were still first‐ or last/senior‐authored by researchers from the United Kingdom and Western Europe, United States, Canada, Australia, and New Zealand, rather than from the country of the study sample being examined. There was little change in these rates for studies published in the 1990s, 2000s, and 2010s.

**Figure 1:**
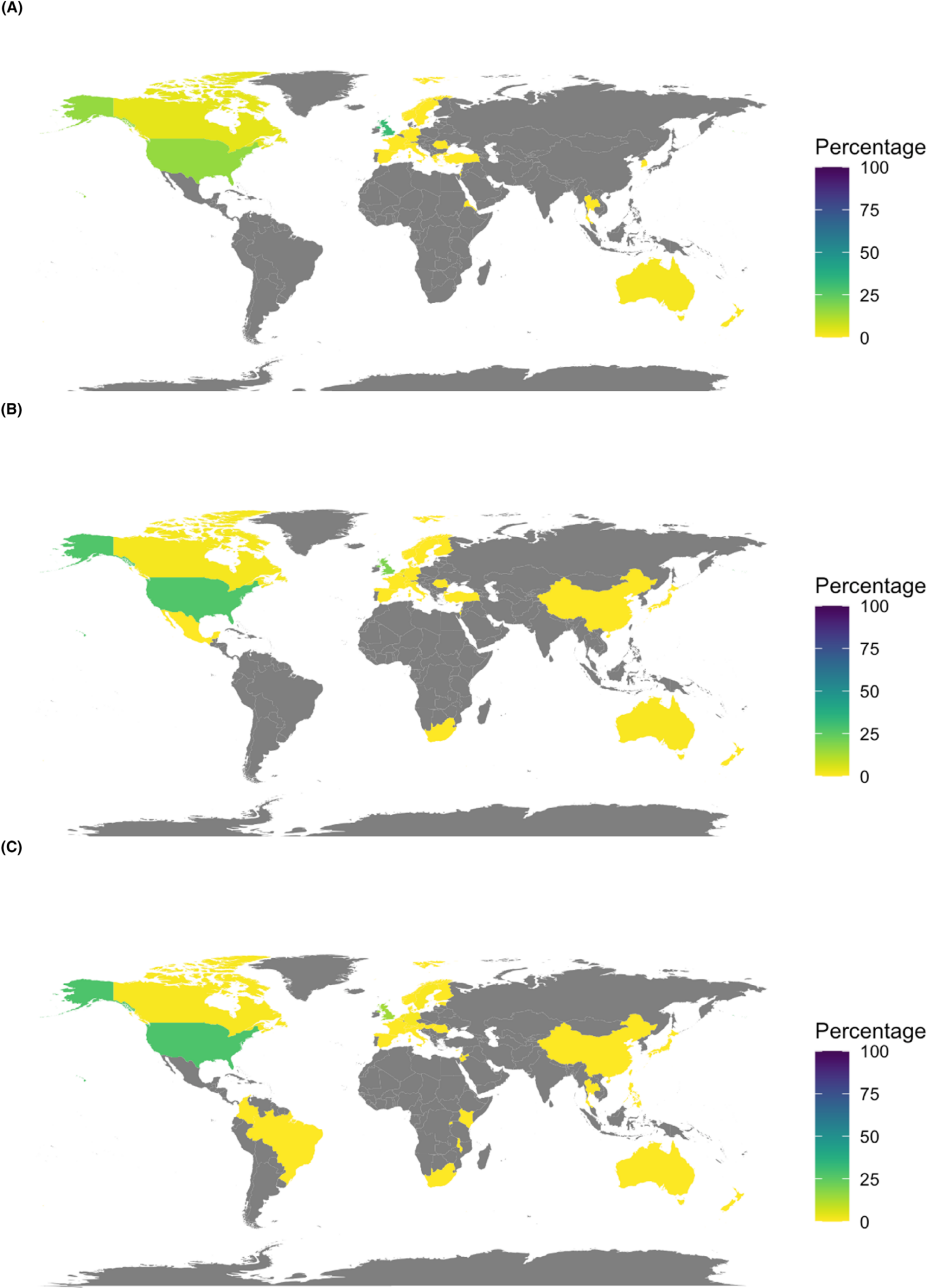
Percentage of samples in each country in studies published in the (A) 1990s, (B) 2000s, and (C) 2010s. Grey indicates there were no samples from that country

We know from the global research that has been conducted on child and adolescent mental health that the prevalence of psychiatric conditions in children and adolescents is generally comparable around the world (e.g., World Health Organization, www.who.int). Critically, the vast majority (85%) of the world population is in Africa, Asia, Central and South America, and the Caribbean, and almost all (90%) of children and adolescents live in low‐ and middle‐income countries. Our understanding of child and adolescent mental health, how it is experienced, expressed, and interpreted, its development and course, risk and protective factors, and how to best prevent and treat psychiatric conditions, is inevitably shaped by who is included in our research and who is conducting it. However, the frameworks and models we use to research child and adolescent mental health have largely been developed with and are centered around a small subset of the global population from countries with high‐income economies^3^ (see also Atilola, 2015; Kieling et al., 2011). Children and adolescents in different parts of the world experience and are exposed to different sociocultural and geopolitical contexts, making it crucial that the frameworks and models we develop are globally informed.^4^ Moreover, in the same way that our child and adolescent mental health research is enriched by the inclusion of samples that reflect the full human population, it is critical to also include researchers that bring varied lived experiences, backgrounds, and identities to their conceptualizations, conduct, and interpretation of the research.^5^

The research that is disseminated is ultimately determined in the journal review and publication process.^6^ *JCPP* is well‐positioned to take the lead in continued and increasing efforts toward enriching the global diversity of our published research. By explicitly valuing globally diverse child and mental health research, *JCPP* can contribute to the science that is conducted and published, while also supporting who is conducting the research, where, and with which samples. When high‐impact journals like *JCPP* make clear that they value globally diverse research—in mission statements, special issues highlighting relevant work, and by spotlighting and promoting relevant publications—and include a globally diverse editorial board and seek out globally representative reviewers, they not only communicate the value of this work, but also improve our science. Understanding the full range of sociocultural and geopolitical contexts and experiential processes that influence child and adolescent well‐being is fundamental for developing and implementing national and international preventions and interventions and public and economic policies that best support children and adolescents worldwide. The human population is increasingly globally interconnected—and *JCPP* has the opportunity to be a leading journal in helping to ensure its research is, too.

## Ethics considerations

Ethics approval is not applicable for this article as it does not include research participants.

## Supporting information


**Appendix S1.** Systematic review.
**Appendix S2.** Literature search.
**Appendix S3.** Data analysis.
**Table S1.** Countries of study samples, in study titles and abstracts, and of study authors in the studies published in the *Journal of Child Psychology and Psychiatry*.
**Table S2.** PRISMA checklist.
**Figure S1.** PRISMA flow diagram of the literature search. A total of 1,976 articles were published in the *Journal of Child Psychology and Psychiatry* during the three sampling time frames. A total of 601 articles were excluded because they were nonempirical or nonhuman. The 1,375 articles included in this systematic review and meta‐analysis reported on 1,601 independent samples.

## Data Availability

The data that support the findings of this study are openly available in OSF at: https://osf.io/e7g6j/?view_only=2b7826c830484851aa1a8db00a9a668b.

## Endnotes

1 An overview of the preregistered systematic review, study coding, data, and analysis code and output are available at https://osf.io/e7g6j/?view_only=2b7826c830484851aa1a8db00a9a668b.

2 See Appendices S1–S3 for an overview of the systematic review, literature search, and data analysis. See Table S2 for the PRISMA checklist and Figure S1 for the PRISMA flow diagram.

3 We recognize that economic resources, even in high‐income countries, may be and often are differentially and inequitably distributed across its population.

4 We want to emphasize that global research on child and adolescent mental health should not be limited to simple comparisons across countries, which too often perpetuate a deficit or deviation viewpoint that privileges a ‘normative’ point of comparison (e.g., Anjum & Aziz, 2024; Atilola, 2015; Coll, Akerman, & Cicchetti, 2000). It is also important to not misinterpret cross‐country differences as reflecting cultural superiority or inferiority. At the same time, although there are differences in sociocultural and geopolitical experiences that may influence child and adolescent mental health that prompt the need for global inclusion in conceptualization and research, we must not forget that there are also fundamental human universalities in child and adolescent development.

5 Recent movements toward large consortium efforts to develop comprehensive datasets of child and adolescent health and well‐being that are made available to the scientific community (e.g., Adolescent Brain Cognitive Development [ABCD] Study; Healthy Brain and Child Development [HBCD] Study; UK Millennium Cohort Study [MCS]) may be particularly beneficial for researchers from countries and institutions that are less highly resourced, as it allows them to work with data they may otherwise have been insufficiently resourced to collect themselves (though note that these consortium studies are often conducted in more highly resourced countries).

6 We recognize that publication is toward the end of a long process throughout which the global diversity of research samples and who is conducting the research may be limited by structural and systemic factors. A critical determinant of the child and mental health research that is conducted and ultimately published is research funding. Although there have been recent efforts (e.g., National Institute of Mental Health Collaborative Hubs for International Research on Mental Health; the Global Coalition for Youth Mental Health), increasing globally diverse child and mental health research will require increased and sustained investment by governments and national and international funding agencies.

## References

[jcpp70163-bib-0001] Anjum, G. , & Aziz, M. (2024). Advancing equity in cross‐cultural psychology: Embracing diverse epistemologies and fostering collaborative practices. Frontiers in Psychology, 15, 1368663. 38638521 10.3389/fpsyg.2024.1368663 PMC11024300

[jcpp70163-bib-0002] Atilola, O. (2015). Cross‐cultural child and adolescent psychiatry research in developing countries. Global Mental Health, 2, e5. 28596853 10.1017/gmh.2015.8 PMC5269637

[jcpp70163-bib-0003] Coll, C.G. , Akerman, A. , & Cicchetti, D. (2000). Cultural influences on developmental processes and outcomes: Implications for the study of development and psychopathology. Development and Psychopathology, 12, 333–356. 11014742 10.1017/s0954579400003059

[jcpp70163-bib-0004] Kieling, C. , Baker‐Henningham, H. , Belfer, M. , Conti, G. , Ertem, I. , Omigbodun, O. , … & Rahman, A. (2011). Child and adolescent mental health worldwide: Evidence for action. The Lancet, 378, 1515–1525. 10.1016/S0140-6736(11)60827-1 22008427

[jcpp70163-bib-0005] Polanczyk, G.V. , Salum, G.A. , Sugaya, L.S. , Caye, A. , & Rohde, L.A. (2015). Annual research review: A meta‐analysis of the worldwide prevalence of mental disorders in children and adolescents. Journal of Child Psychology and Psychiatry, 56, 345–365. 25649325 10.1111/jcpp.12381

[jcpp70163-bib-0006] Sonuga‐Barke, E.J.S. (2012). Editorial: “Holy grail” or “Siren's song”?: The dangers for the field of child psychology and psychiatry of over‐focusing on the journal impact factor. Journal of Child Psychology and Psychiatry, 53, 915–917. 22924400 10.1111/j.1469-7610.2012.02612.x

[jcpp70163-bib-0007] Sonuga‐Barke, E.J.S. (2014). Editorial: Building global science capacity in child psychology and psychiatry—Between the etic and emic of cross‐cultural enquiry. Journal of Child Psychology and Psychiatry, 55, 301–303. 24661062 10.1111/jcpp.12234

