# Supplementary material for: How global is our research on child and adolescent mental health? A worldwide concentration and imbalance – and an opportunity for JCPP to take the lead in disseminating globally diverse research
Source: J Child Psychol Psychiatry. 2026 Apr 27;67(9):1614–7. doi: 10.1111/jcpp.70163 (PMC13450272; doi:10.1111/jcpp.70163)
Supplement: Supplementary file 1 [file JCPP-67-1614-s001.docx]

**Editorial Perspective: How Global Is Our Research on Child and Adolescent Mental Health? A Worldwide Concentration and Imbalance—and an Opportunity for *JCPP* to Take the Lead in Disseminating Globally Diverse Research**

**Supporting Information**

**Appendix S1. Systematic Review**

In this systematic review, we considered how global our child and adolescent mental health research is in terms of where in the world study samples and authors are from in studies published over the past three decades (in the 1990s, 2000s, and 2010s) in the *Journal of Child Psychology and Psychiatry*. We considered (1) the countries in which research has been conducted, (2) whether the location of the study sample was noted in the study title or abstract^[1]^, and (3) countries of study authors, as well as whether there have been changes over time.

**Methods**

This systematic review and meta-analysis conformed to PRISMA guidelines (see Table S2 for the PRISMA checklist). Study coding and data and analysis code and output are available at https://osf.io/e7g6j/?view_only=2b7826c830484851aa1a8db00a9a668b. Study analyses were preregistered and the two minor deviations from the preregistration are noted in Footnote 1 and Footnote 2.

**Appendix S2. Literature Search**

**Database search.** A systematic search in the PsycINFO and MEDLINE databases^[2]^ identified all articles published in the *Journal of Child Psychiatry and Psychology* in three sampling time frames in the 1990s (January 1, 1995, to December 31, 1999), 2000s (January 1, 2005, to December 31, 2009), and 2010s (January 1, 2015, to December 31, 2019). These 5-year time frames were selected to provide a sampling of the total number of publications over the past three decades (including the most recent publications at the time the systematic search was conducted in 2020).

**Inclusion and exclusion criteria.** Empirical studies were included if they reported on a sample of human participants. Studies were excluded if they were nonempirical (e.g., reviews, editorials, commentaries, retractions) or not conducted in a human sample (e.g., simulations). See Figure S1 for the PRISMA flow diagram for the literature search.

**Study coding.** All included studies were coded for (1) the country^[3],[4]^ of the sample, (2)(a) whether the country (or location from which country could be coded) of the sample was noted in the title or abstract, and, if so, (2)(b) the country that was noted, and (3) the country of study authors (based on their institutional affiliation). All studies were coded and second coded by trained research assistants under the supervision of the first author, and any discrepancies in coding were identified and resolved by discussion and consensus. Preparation of the final dataset was completed by the first author.

**Appendix S3. Data Analysis**

We first examined the proportion of study samples from each of the countries represented in the past three decades and whether there were changes over time. Next, we examined whether the location of the sample was noted in the title or abstract, and the proportion of studies for each country that did so. Finally, we examined the proportion of countries study authors were from. All analyses were conducted using the “metaprop” function in the “meta” package (Schwarzer, 2022) in R (version 4.2.1), so that, for each set of studies (*k* is the number of studies), each analysis yielded an overall mean (pooled) point estimate (presented as percentages) with a 95% confidence interval (CI) within each sampling time frame. Although it is possible to present simple percentages for proportions, we took this meta-analytic approach because it yields both overall percentages and 95% CIs that provide an indication of the precision and robustness of effects, and allows for the statistical comparison (using the *Q_B_* statistic) of overall percentages across sampling time frames. The significance level was set at *p* < .050 and *p* values are presented to 3 digits to allow researchers to consider different significance levels.

**Table S1.** Countries of Study Samples, in Study Titles and Abstracts, and of Study Authors in the Studies Published in the *Journal of Child Psychology and Psychiatry*

|  | **1990s** | | **2000s** | | **2010s** | |  |  |
| --- | --- | --- | --- | --- | --- | --- | --- | --- |
|  |  |  |  |  |  |  |  |  |
|  | ***n*/*k*** | **% (95% CI)** | ***n*/*k*** | **% (95% CI)** | ***n*/*k*** | **% (95% CI)** |  |  |
|  |  |  |  |  |  |  |  |  |
| **Country of study samples** |  |  |  |  |  |  |  |  |
| **Afghanistan** | 0/398 | 0.00% (0.00%, 1.11%) | 0/593 | 0.00% (0.00%, 0.77%) | 2/610 | 0.00% (0.00%, 0.80%) |  |  |
|  |  |  |  |  | *Q_B_*(2) = 0.00, *p* = .998 | |  |  |
| **Australia** | 21/398 | 1.18% (0.00%, 4.05%) | 27/593 | 0.66% (0.00%, 2.58%) | 26/610 | 0.31% (0.00%, 1.86%) |  |  |
|  |  |  |  |  | *Q_B_*(2) = 0.63, *p* = .729 | |  |  |
| **Austria** | 0/398 | 0.00% (0.00%, 1.11%) | 1/593 | 0.00% (0.00%, 0.82%) | 2/610 | 0.01% (0.00%, 0.86%) |  |  |
|  |  |  |  |  | *Q_B_*(2) = 0.01, *p* = .995 | |  |  |
| **Bangladesh** | 0/398 | 0.00% (0.00%, 1.11%) | 1/593 | 0.00% (0.00%, 0.82%) | 0/610 | 0.00% (0.00%, 0.74%) |  |  |
|  |  |  |  |  | *Q_B_*(2) = 0.00, *p* = .998 | |  |  |
| **Belarus** | 1/398 | 0.00% (0.00%, 1.22%) | 0/593 | 0.00% (0.00%, 0.77%) | 0/610 | 0.00% (0.00%, 0.74%) |  |  |
|  |  |  |  |  | *Q_B_*(2) = 0.01, *p* = .997 | |  |  |
| **Belgium** | 1/398 | 0.00% (0.00%, 1.22%) | 7/593 | 0.07% (0.00%, 1.22%) | 5/610 | 0.03% (0.00%, 1.05%) |  |  |
|  |  |  |  |  | *Q_B_*(2) = 0.08, *p* = .923 | |  |  |
| **Brazil** | 0/398 | 0.00% (0.00%, 1.11%) | 1/593 | 0.00% (0.00%, 0.82%) | 6/610 | 0.03% (0.00%, 1.05%) |  |  |
|  |  |  |  |  | *Q_B_*(2) = 0.07, *p* = .963 | |  |  |
| **Bulgaria** | 0/398 | - | 0/593 | - | 0/610 | - |  |  |
|  |  |  |  |  |  | |  |  |
| **Burkina Faso** | 0/398 | 0.00% (0.00%, 1.11%) | 0/593 | 0.00% (0.00%, 0.77%) | 1/610 | 0.00% (0.00%, 0.80%) |  |  |
|  |  |  |  |  | *Q_B_*(2) = 0.00, *p* = .998 | |  |  |
| **Burundi** | 0/398 | 0.00% (0.00%, 1.11%) | 1/593 | 0.00% (0.00%, 0.82%) | 0/610 | 0.00% (0.00%, 0.74%) |  |  |
|  |  |  |  |  | *Q_B_*(2) = 0.00, *p* = .998 | |  |  |
| **Canada** | 39/398 | 3.66% (0.95%, 7.59%) | 41/593 | 1.53% (0.17%, 3.96%) | 31/610 | 0.97% (0.02%, 3.07%) |  |  |
|  |  |  |  |  | *Q_B_*(2) = 2.33, *p* = .312 | |  |  |
| **Chile** | 0/398 | - | 0/593 | - | 0/610 | - |  |  |
|  |  |  |  |  |  | |  | |
| **China** | 2/398 | 0.00% (0.00%, 1.32%) | 3/593 | 0.01% (0.00%, 0.95%) | 9/610 | 0.10% (0.00%, 1.32%) |  |  |
|  |  |  |  |  | *Q_B_*(2) = 0.13, *p* = .936 | |  |  |
| **Colombia** | 0/398 | 0.00% (0.00%, 1.11%) | 0/593 | 0.00% (0.00%, 0.77%) | 3/610 | 0.01% (0.00%, 0.86%) |  |  |
|  |  |  |  |  | *Q_B_*(2) = 0.02, *p* = .993 | |  |  |
| **Cuba** | 0/398 | - | 0/593 | - | 0/610 | - |  |  |
|  |  |  |  |  |  | |  | |
| **Cyprus** | 1/398 | 0.00% (0.00%, 1.22%) | 0/593 | 0.00% (0.00%, 0.77%) | 2/610 | 0.01% (0.00%, 0.86%) |  |  |
|  |  |  |  |  | *Q_B_*(2) = 0.01, *p* = .993 | |  |  |
| **Czech Republic** | 0/398 | - | 0/593 | - | 0/610 | - |  |  |
|  |  |  |  |  |  | |  |  |
| **Denmark** | 1/398 | 0.00% (0.00%, 1.22%) | 6/593 | 0.05% (0.00%, 1.15%) | 6/610 | 0.05% (0.00%, 1.11%) |  |  |
|  |  |  |  |  | *Q_B_*(2) = 0.06, *p* = .972 | |  |  |
| **Eritrea** | 2/398 | 0.01% (0.00%, 1.32%) | 0/593 | 0.00% (0.00%, 0.77%) | 0/610 | 0.00% (0.00%, 0.74%) |  |  |
|  |  |  |  |  | *Q_B_*(2) = 0.03, *p* = .986 | |  |  |
| **Estonia** | 0/398 | 0.00% (0.00%, 1.11%) | 0/593 | 0.00% (0.00%, 0.77%) | 2/610 | 0.01% (0.00%, 0.86%) |  |  |
|  |  |  |  |  | *Q_B_*(2) = 0.02, *p* = .993 | |  |  |
| **Ethiopia** | 0/398 | 0.00% (0.00%, 1.11%) | 1/593 | 0.00% (0.00%, 0.82%) | 0/610 | 0.00% (0.00%, 0.74%) |  |  |
|  |  |  |  |  | *Q_B_*(2) = 0.00, *p* = .998 | |  |  |
| **Finland** | 1/398 | 0.00% (0.00%, 1.22%) | 7/593 | 0.07% (0.00%, 1.22%) | 5/610 | 0.02% (0.00%, 0.98%) |  |  |
|  |  |  |  |  | *Q_B_*(2) = 0.08, *p* = .961 | |  |  |
| **France** | 2/398 | 0.01% (0.00%, 1.32%) | 4/593 | 0.01% (0.00%, 0.95%) | 7/610 | 0.05% (0.00%, 1.11%) |  |  |
|  |  |  |  |  | *Q_B_*(2) = 0.03, *p* = .984 | |  |  |
| **Germany** | 9/398 | 0.14% (0.00%, 1.92%) | 13/593 | 0.22% (0.00%, 1.68%) | 32/610 | 1.11% (0.04%, 3.27%) |  |  |
|  |  |  |  |  | *Q_B_*(2) = 1.33, *p* = .515 | |  |  |
| **Ghana** | 0/398 | 0.00% (0.00%, 1.11%) | 0/593 | 0.00% (0.00%, 0.77%) | 1/610 | 0.00% (0.00%, 0.81%) |  |  |
|  |  |  |  |  | *Q_B_*(2) = 0.00, *p* = .998 | |  |  |
| **Greece** | 3/398 | 0.03% (0.00%, 1.44%) | 1/593 | 0.00% (0.00%, 0.82%) | 3/610 | 0.00% (0.00%, 0.80%) |  |  |
|  |  |  |  |  | *Q_B_*(2) = 0.04, *p* = .981 | |  |  |
| **Hong Kong** | 5/398 | 0.05% (0.00%, 1.55%) | 6/593 | 0.05% (0.00%, 1.55%) | 0/593 | 0.00% (0.00%, 0.74%) |  |  |
|  |  |  |  |  | *Q_B_*(2) = 0.14, *p* = .931 | |  |  |
| **Hungary** | 0/398 | 0.00% (0.00%, 1.11%) | 1/593 | 0.00% (0.00%, 0.82%) | 3/610 | 0.01% (0.00%, 0.92%) |  |  |
|  |  |  |  |  | *Q_B_*(2) = 0.02, *p* = .981 | |  |  |
| **India** | 1/398 | 0.00% (0.00%, 1.22%) | 0/593 | 0.00% (0.00%, 0.77%) | 0/610 | 0.00% (0.00%, 0.74%) |  |  |
|  |  |  |  |  | *Q_B_*(2) = 0.01, *p* = .997 | |  |  |
| **Indonesia** | 0/398 | 0.00% (0.00%, 1.11%) | 1/593 | 0.00% (0.00%, 0.82%) | 0/610 | 0.00% (0.00%, 0.74%) |  |  |
|  |  |  |  |  | *Q_B_*(2) = 0.00, *p* = .998 | |  |  |
| **Iran** | 0/398 | - | 0/593 | - | 0/610 | - |  |  |
|  |  |  |  |  |  | |  |  |
| **Israel** | 8/398 | 0.19% (0.00%, 2.05%) | 9/593 | 0.11% (0.00%, 1.37%) | 6/610 | 0.03% (0.00%, 1.05%) |  |  |
|  |  |  |  |  | *Q_B_*(2) = 0.13, *p* = .935 | |  |  |
| **Italy** | 2/398 | 0.01% (0.00%, 1.32%) | 6/593 | 0.03% (0.00%, 1.08%) | 7/610 | 0.06% (0.00%, 1.18%) |  |  |
|  |  |  |  |  | *Q_B_*(2) = 0.04, *p* = .982 | |  |  |
| **Jamaica** | 1/398 | 0.00% (0.00%, 1.22%) | 0/593 | 0.00% (0.00%, 0.77%) | 0/610 | 0.00% (0.0%, 0.75%) |  |  |
|  |  |  |  |  | *Q_B_*(2) = 0.01, *p* = .997 | |  |  |
| **Japan** | 0/398 | 0.00% (0.00%, 1.11%) | 3/593 | 0.01% (0.00%, 0.95%) | 2/610 | 0.01% (0.00%, 0.86%) |  |  |
|  |  |  |  |  | *Q_B_*(2) = 0.2, *p* = .989 | |  |  |
| **Jordan** | 0/398 | 0.00% (0.00%, 1.11%) | 0/593 | 0.00% (0.00%, 0.77%) | 3/610 | 0.01% (0.00%, 0.92%) |  |  |
|  |  |  |  |  | *Q_B_*(2) = 0.03, *p* = .983 | |  |  |
| **Kenya** | 0/398 | 0.00% (0.00%, 1.11%) | 0/593 | 0.00% (0.00%, 0.77%) | 4/610 | 0.02% (0.00%, 0.98%) |  |  |
|  |  |  |  |  | *Q_B_*(2) = 0.06, *p* = .970 | |  |  |
| **Korea** | 3/398 | 0.03% (0.00%, 1.44%) | 0/593 | 0.00% (0.00%, 0.77%) | 0/610 | 0.00% (0.00%, 0.74%) |  |  |
|  |  |  |  |  | *Q_B_*(2) = 0.06, *p* = .969 | |  |  |
| **Malawi** | 0/398 | 0.00% (0.00%, 1.11%) | 0/593 | 0.00% (0.00%, 0.77%) | 2/610 | 0.01% (0.00%, 0.86%) |  |  |
|  |  |  |  |  | *Q_B_*(2) = 0.02, *p* = .992 | |  |  |
| **Mauritius** | 0/398 | 0.00% (0.00%, 1.11%) | 0/593 | 0.00% (0.00%, 0.77%) | 1/610 | 0.00% (0.00%, 0.80%) |  |  |
|  |  |  |  |  | *Q_B_*(2) = 0.00, *p* = .998 | |  |  |
| **Mexico** | 0/398 | 0.00% (0.00%, 1.11%) | 2/593 | 0.01% (0.00%, 0.89%) | 0/610 | 0.00% (0.00%, 0.74%) |  |  |
|  |  |  |  |  | *Q_B_*(2) = 0.02, *p* = .992 | |  |  |
| **Netherlands** | 20/398 | 0.98% (0.00%, 3.71%) | 52/593 | 2.90% (0.85%, 5.83%) | 45/610 | 1.77% (0.27%, 4.26%) |  |  |
|  |  |  |  |  | *Q_B_*(2) = 1.33, *p* = .515 | |  |  |
| **New Zealand** | 14/398 | 0.55% (0.00%, 2.91%) | 11/593 | 0.07% (0.00%, 1.22%) | 9/610 | 0.10% (0.00%, 1.32%) |  |  |
|  |  |  |  |  | *Q_B_*(2) = 0.52, *p* = .764 | |  |  |
| **Nicaragua** | 0/398 | 0.00% (0.00%, 1.11%) | 1/593 | 0.00% (0.00%, 0.82%) | 0/610 | 0.00% (0.00%, 0.74%)  *Q_B_*(2) = 0.00, *p* = .998 |  |  |
| **Nigeria** | 1/398 | 0.00% (0.00%, 1.22%) | 0/593 | 0.00% (0.00%, 0.77%) | 0/610 | 0.00% (0.0%, 0.75%) |  |  |
|  |  |  |  |  | *Q_B_*(2) = 0.01, *p* = .997 | |  |  |
| **Norway** | 3/398 | 0.03% (0.00%, 1.44%) | 10/593 | 0.13% (0.00%, 1.44%) | 10/610 | 0.13% (0.00%, 1.39%) |  |  |
|  |  |  |  |  | *Q_B_*(2) = 0.09, *p* = .995 | |  |  |
| **Palestine** | 1/398 | 0.00% (0.00%, 1.22%) | 1/593 | 0.00% (0.00%, 0.82%) | 0/610 | 0.00% (0.00%, 0.74%) |  |  |
|  |  |  |  |  | *Q_B_*(2) = 0.01, *p* = .997 | |  |  |
| **Philippines** | 0/398 | 0.00% (0.00%, 1.11%) | 0/593 | 0.00% (0.00%, 0.77%) | 3/610 | 0.01% (0.00%, 0.92%) |  |  |
|  |  |  |  |  | *Q_B_*(2) = 0.03, *p* = .983 | |  |  |
| **Poland** | 0/398 | 0.00% (0.00%, 1.11%) | 1/593 | 0.00% (0.00%, 0.82%) | 1/610 | 0.00% (0.00%, 0.80%) |  |  |
|  |  |  |  |  | *Q_B_*(2) = 0.00, *p* = .999 | |  |  |
| **Portugal** | 1/398 | 0.00% (0.00%, 1.22%) | 0/593 | 0.00% (0.00%, 0.77%) | 0/610 | 0.00% (0.00%, 0.74%) |  |  |
|  |  |  |  |  | *Q_B_*(2) = 0.01, *p* = .997 | |  |  |
| **Puerto Rico** | 1/398 | 0.00% (0.00%, 1.22%) | 5/593 | 0.03% (0.00%, 1.08%) | 1/610 | 0.00% (0.00%, 0.80%) |  |  |
|  |  |  |  |  | *Q_B_*(2) = 0.06, *p* = .971 | |  |  |
| **Qatar** | 0/398 | 0.00% (0.00%, 1.11%) | 0/593 | 0.00% (0.00%, 0.77%) | 2/610 | 0.00% (0.00%, 0.80%) |  |  |
|  |  |  |  |  | *Q_B_*(2) = 0.00, *p* = .998 | |  |  |
| **Romania** | 2/398 | 0.01% (0.00%, 1.32%) | 6/593 | 0.05% (0.00%, 1.15%) | 5/610 | 0.03% (0.00%, 1.05%) |  |  |
|  |  |  |  |  | *Q_B_*(2) = 0.02, *p* = .987 | |  |  |
| **Russia** | 1/398 | 0.00% (0.00%, 1.22%) | 0/593 | 0.00% (0.00%, 0.77%) | 0/610 | 0.00% (0.00%, 0.74%) |  |  |
|  |  |  |  |  | *Q_B_*(2) = 0.01, *p* = .997 | |  |  |
| **Rwanda** | 0/398 | 0.00% (0.00%, 1.11%) | 0/593 | 0.00% (0.00%, 0.77%) | 2/610 | 0.01% (0.00%, 0.86%) |  |  |
|  |  |  |  |  | *Q_B_*(2) = 0.02, *p* = .993 | |  |  |
| **Sierra Leone** | 0/398 | 0.00% (0.00%, 1.11%) | 0/593 | 0.00% (0.00%, 0.77%) | 4/610 | 0.00% (0.00%, 0.80%) |  |  |
|  |  |  |  |  | *Q_B_*(2) = 0.00, *p* = .998 | |  |  |
| **Singapore** | 0/398 | - | 0/593 | - | 0/610 | - |  |  |
|  |  |  |  |  |  | |  |  |
| **Slovenia** | 0/398 | 0.00% (0.00%, 1.11%) | 0/593 | 0.00% (0.00%, 0.77%) | 2/610 | 0.01% (0.00%, 0.86%) |  |  |
|  |  |  |  |  | *Q_B_*(2) = 0.02, *p* = .993 | |  |  |
| **South Africa** | 0/398 | 0.00% (0.00%, 1.11%) | 3/593 | 0.01% (0.00%, 0.89%) | 3/610 | 0.01% (0.00%, 0.86%) |  |  |
|  |  |  |  |  | *Q_B_*(2) = 0.01, *p* = .997 | |  |  |
| **Spain** | 4/398 | 0.03% (0.00%, 1.44%) | 4/593 | 0.02% (0.00%, 1.01%) | 6/610 | 0.05% (0.00%, 1.11%) |  |  |
|  |  |  |  |  | *Q_B_*(2) = 0.01, *p* = .994 | |  |  |
| **Sri Lanka** | 1/398 | 0.00% (0.00%, 1.11%) | 1/593 | 0.00% (0.00%, 0.82%) | 0/610 | 0.00% (0.00%, 0.74%) |  |  |
|  |  |  |  |  | *Q_B_*(2) = 0.01, *p* = .997 | |  |  |
| **Sudan** | 1/398 | 0.00% (0.00%, 1.22%) | 1/593 | 0.00% (0.00%, 0.82%) | 0/610 | 0.00% (0.00%, 0.74%) |  |  |
|  |  |  |  |  | *Q_B_*(2) = 0.01, *p* = .997 | |  |  |
| **Sweden** | 7/398 | 0.14% (0.00%, 1.92%) | 11/593 | 0.11% (0.00%, 1.37%) | 32/610 | 0.97% (0.02%, 3.07%) |  |  |
|  |  |  |  |  | *Q_B_*(2) = 1.29, *p* = .524 | |  |  |
| **Switzerland** | 2/398 | 0.01% (0.00%, 1.32%) | 9/593 | 0.11% (0.00%, 1.37%) | 3/610 | 0.01% (0.00%, 0.92%) |  |  |
|  |  |  |  |  | *Q_B_*(2) = 0.14, *p* = .930 | |  |  |
| **Syria** | 0/398 | 0.00% (0.00%, 1.11%) | 0/593 | 0.00% (0.00%, 0.77%) | 1/610 | 0.00% (0.00%, 0.80%) |  |  |
|  |  |  |  |  | *Q_B_*(2) = 0.00, *p* = .998 | |  |  |
| **Taiwan** | 0/398 | 0.00% (0.00%, 1.11%) | 0/593 | 0.00% (0.00%, 0.77%) | 3/610 | 0.01% (0.00%, 0.92%) |  |  |
|  |  |  |  |  | *Q_B_*(2) = 0.03, *p* = .983 | |  |  |
| **Thailand** | 1/398 | 0.00% (0.00%, 1.22%) | 0/593 | 0.00% (0.00%, 0.77%) | 3/610 | 0.01% (0.00%, 0.92%) |  |  |
|  |  |  |  |  | *Q_B_*(2) = 0.03, *p* = .987 | |  |  |
| **Turkey** | 2/398 | 0.01% (0.00%, 1.32%) | 2/593 | 0.01% (0.00%, 0.89%) | 0/610 | 0.00% (0.00%, 0.74%) |  |  |
|  |  |  |  |  | *Q_B_*(2) = 0.03, *p* = .988 | |  |  |
| **Uganda** | 1/398 | 0.00% (0.00%, 1.22%) | 0/593 | 0.00% (0.00%, 0.77%) | 1/610 | 0.00% (0.00%, 0.80%) |  |  |
|  |  |  |  |  | *Q_B_*(2) = 0.01, *p* = .997 | |  |  |
| **United Kingdom** | 148/398 | 31.83% (25.34%, 38.56%) | 166/593 | 19.32% (14.65%, 24.31%) | 139/610 | 13.78% (9.66%, 18.32%) |  |  |
|  |  |  |  |  | *Q_B_*(2) = 20.51, *p* < .001 | |  |  |
| **England** | 121/398 | 23.29% (17.30%, 29.66%) | 102/593 | 8.58% (5.10%, 11.22%) | 78/610 | 5.54% (2.67%, 8.95%) |  |  |
|  |  |  |  |  | *Q_B_*(2) = 28.19, *p* < .001 | |  |  |
| **Ireland** | 1/398 | 0.00% (0.00%, 1.22%) | 7/593 | 0.05% (0.00%, 1.15%) | 7/610 | 0.06% (0.00%, 1.18%) |  |  |
|  |  |  |  |  | *Q_B_*(2) = 0.08, *p* = .962 | |  |  |
| **Isle of Wight** | 0/398 | - | 0/593 | - | 0/610 | - |  |  |
|  |  |  |  |  |  | |  |  |
| **Scotland** | 7/398 | 0.14% (0.00%, 1.92%) | 6/593 | 0.03% (0.00%, 1.08%) | 4/610 | 0.02% (0.00%, 0.98%) |  |  |
|  |  |  |  |  | *Q_B_*(2) = 0.12, *p* = .943 | |  |  |
| **Wales** | 4/398 | 0.05% (0.00%, 1.55%) | 4/593 | 0.02% (0.00%, 1.01%) | 4/610 | 0.01% (0.00%, 0.92%) |  |  |
|  |  |  |  |  | *Q_B_*(2) = 0.00, *p* = .998 | |  |  |
| **United States of America** | 97/398 | 15.74% (10.44%, 21.57%) | 217/593 | 27.29% (22.14%, 32.65%) | 211/610 | 27.99% (22.26%, 33.05%) |  |  |
|  |  |  |  |  | *Q_B_*(2) = 11.02, *p* = .004 | |  |  |
| **Zambia** | 0/398 | 0.00% (0.00%, 1.11%) | 0/593 | 0.00% (0.00%, 0.77%) | 1/610 | 0.00% (0.00%, 0.80%) |  |  |
|  |  |  |  |  | *Q_B_*(2) = 0.00, *p* = .998 | |  |  |
| **Countries given in study titles/abstracts** | |  |  |  |  |  |  |  |
| **Afghanistan** | 0/69 | 0.00% (0.00%, 5.43%) | 0/120 | 0.00% (0.00%, 3.33%) | 1/143 | 0.02% (0.00%, 3.28%) |  |  |
|  |  |  |  |  | *Q_B_*(2) = 0.01, *p* = .992 | |  |  |
| **Australia** | 4/69 | 1.14% (0.00%, 10.50%) | 3/120 | 0.29% (0.00%, 5.07%) | 5/143 | 0.54% (0.00%, 5.24%) |  |  |
|  |  |  |  |  | *Q_B_*(2) = 0.18, *p* = .915 | |  |  |
| **Austria** | 0/69 | - | 0/120 | - | 0/143 | - |  |  |
|  |  |  |  |  |  | |  |  |
| **Bangladesh** | 0/69 | 0.00% (0.00%, 5.43%) | 1/120 | 0.03% (0.00%, 3.88%) | 0/143 | 0.00% (0.00%, 2.85%) |  |  |
|  |  |  |  |  | *Q_B_*(2) = 0.02, *p* = .990 | |  |  |
| **Belarus** | 1/69 | 0.10% (0.00%, 6.59%) | 0/120 | 0.00% (0.00%, 3.33%) | 0/143 | 0.00% (0.00%, 2.85%) |  |  |
|  |  |  |  |  | *Q_B_*(2) = 0.04, *p* = .979 | |  |  |
| **Belgium** | 0/69 | 0.00% (0.00%, 5.43%) | 0/120 | 0.00% (0.00%, 3.33%) | 1/143 | 0.02% (0.00%, 3.28%) |  |  |
|  |  |  |  |  | *Q_B_*(2) = 0.01, *p* = .993 | |  |  |
| **Brazil** | 0/69 | 0.00% (0.00%, 5.43%) | 1/120 | 0.03% (0.00%, 3.88%) | 2/143 | 0.09% (0.00%, 3.74%) |  |  |
|  |  |  |  |  | *Q_B_*(2) = 0.03, *p* = .983 | |  |  |
| **Bulgaria** | 0/69 | - | 0/120 | - | 0/143 | - |  |  |
|  |  |  |  |  |  | |  |  |
| **Burkina Faso** | 0/69 | 0.00% (0.00%, 5.43%) | 0/120 | 0.00% (0.00%, 3.33%) | 1/143 | 0.02% (0.00%, 3.28%) |  |  |
|  |  |  |  |  | *Q_B_*(2) = 0.01, *p* = .993 | |  |  |
| **Burundi** | 0/69 | 0.00% (0.00%, 5.43%) | 1/120 | 0.03% (0.00%, 3.88%) | 0/143 | 0.00% (0.00%, 2.85%) |  |  |
|  |  |  |  |  | *Q_B_*(2) = 0.02, *p* = .990 | |  |  |
| **Canada** | 7/69 | 3.89% (0.00%, 14.95%) | 4/120 | 0.50% (0.00%, 5.71%) | 4/143 | 0.36% (0.00%, 4.72%) |  |  |
|  |  |  |  |  | *Q_B_*(2) = 1.03, *p* = .598 | |  |  |
| **Chile** | 0/69 | - | 0/120 | - | 0/143 | - |  |  |
|  |  |  |  |  |  | |  |  |
| **China** | 2/69 | 0.38% (0.00%, 7.82%) | 4/120 | 0.50% (0.00%, 5.71%) | 3/143 | 0.20% (0.00%, 4.22%) |  |  |
|  |  |  |  |  | *Q_B_*(2) = 0.04, *p* = .981 | |  |  |
| **Colombia** | 0/69 | 0.00% (0.00%, 5.43%) | 0/120 | 0.00% (0.00%, 3.33%) | 2/143 | 0.09% (0.00%, 3.74%) |  |  |
|  |  |  |  |  | *Q_B_*(2) = 0.06, *p* = .971 | |  |  |
| **Cuba** | 0/69 | - | 0/120 | - | 0/143 | - |  |  |
|  |  |  |  |  |  | |  |  |
| **Cyprus** | 1/69 | 0.10% (0.00%, 6.59%) | 0/120 | 0.00% (0.00%, 3.33 %) | 2/143 | 0.09% (0.00%, 3.74%) |  |  |
|  |  |  |  |  | *Q_B_*(2) = 0.06, *p* = .972 | |  |  |
| **Czech Republic** | 0/69 | - | 0/120 | - | 0/143 | - |  |  |
|  |  |  |  |  |  | |  |  |
| **Denmark** | 0/69 | 0.00% (0.00%, 5.43%) | 5/120 | 0.76% (0.00%, 6.38%) | 5/143 | 0.54% (0.00%, 5.24%) |  |  |
|  |  |  |  |  | *Q_B_*(2) = 0.3, *p* = .859 | |  |  |
| **Eritrea** | 2/69 | 0.38% (0.05%, 7.82%) | 0/120 | 0.00% (0.00%, 3.33%) | 0/143 | 0.00% (0.00%, 2.85%) |  |  |
|  |  |  |  |  | *Q_B_*(2) = 0.17, *p* = .919 | |  |  |
| **Estonia** | 0/69 | - | 0/120 | - | 0/143 | - |  |  |
|  |  |  |  |  |  | |  |  |
| **Ethiopia** | 0/69 | - | 0/120 | - | 0/143 | - |  |  |
|  |  |  |  |  |  | |  |  |
| **Finland** | 0/69 | 0.00% (0.00%, 5.43%) | 6/120 | 1.07% (0.00%, 7.08%) | 4/143 | 0.36% (0.00%, 4.72%) |  |  |
|  |  |  |  |  | *Q_B_*(2) = 0.41, *p* = .815 | |  |  |
| **France** | 0/69 | 0.00% (0.00%, 5.43%) | 0/120 | 0.00% (0.00%, 3.33%) | 2/143 | 0.09% (0.00%, 3.74%) |  |  |
|  |  |  |  |  | *Q_B_*(2) = 0.06, *p* = .971 | |  |  |
| **Germany** | 1/69 | 0.00% (0.00%, 6.59%) | 2/120 | 0.13% (0.00%, 4.46%) | 7/143 | 1.03% (0.00%, 6.32%) |  |  |
|  |  |  |  |  | *Q_B_*(2) = 0.33, *p* = .848 | |  |  |
| **Ghana** | 0/69 | 0.00% (0.00%, 5.43%) | 0/120 | 0.00% (0.00%, 3.33%) | 1/143 | 0.02% (0.00%, 3.28%) |  |  |
|  |  |  |  |  | *Q_B_*(2) = 0.01, *p* = .993 | |  |  |
| **Greece** | 3/69 | 0.82% (0.00%, 9.13%) | 0/120 | 0.00% (0.00%, 3.33%) | 2/143 | 0.09% (0.00%, 3.74%) |  |  |
|  |  |  |  |  | *Q_B_*(2) = 0.31, *p* = .856 | |  |  |
| **Hong Kong** | 2/69 | 0.38% (0.00%, 7.82%) | 2/120 | 0.13% (0.00%, 4.46%) | 0/143 0.00% (0.00%, 2.85%) | |  |  |
|  |  |  |  |  | *Q_B_*(2) = 0.16, *p* = .923 | |  |  |
| **Hungary** | 0/69 | - | 0/120 | - | 0/143 | - |  |  |
|  |  |  |  |  |  | |  |  |
| **India** | 1/69 | 0.00% (0.00%, 6.59%) | 0/120 | 0.00% (0.00%, 3.33%) | 0/143 | 0.00% (0.00%, 2.85%) |  |  |
|  |  |  |  |  | *Q_B_*(2) = 0.04, *p* = .979 | |  |  |
| **Indonesia** | 0/69 | 0.00% (0.00%, 5.43%) | 1/120 | 0.03% (0.00%, 3.88%) | 0/143 | 0.00% (0.00%, 2.85%) |  |  |
|  |  |  |  |  | *Q_B_*(2) = 0.02, *p* = .990 | |  |  |
| **Iran** | 0/69 | - | 0/120 | - | 0/143 | - |  |  |
|  |  |  |  |  |  | |  |  |
| **Israel** | 2/69 | 0.38% (0.00%, 7.28%) | 2/120 | 0.13% (0.00%, 4.46%) | 0/143 | 0.00% (0.00%, 2.85%) |  |  |
|  |  |  |  |  | *Q_B_*(2) = 0.16, *p* = .923 | |  |  |
| **Italy** | 0/69 | 0.00% (0.00%, 5.43%) | 2/120 | 0.13% (0.00%, 4.46%) | 2/143 | 0.09% (0.00%, 3.74%) |  |  |
|  |  |  |  |  | *Q_B_*(2) = 0.05, *p* = .976 | |  |  |
| **Jamaica** | 1/69 | 0.00% (0.00%, 6.59%) | 0/120 | 0.00% (0.00%, 3.33%) | 0/143 | 0.00% (0.00%, 2.85%) |  |  |
|  |  |  |  |  | *Q_B_*(2) = 0.04, *p* = .979 | |  |  |
| **Japan** | 0/69 | 0.00% (0.00%, 5.43%) | 2/120 | 0.13% (0.00%, 4.46%) | 0/143 | 0.00% (0.00%, 2.85%) |  |  |
|  |  |  |  |  | *Q_B_*(2) = 0.08, *p* = .961 | |  |  |
| **Jordan** | 0/69 | 0.00% (0.00%, 5.43%) | 0/120 | 0.00% (0.00%, 3.33%) | 2/143 | 0.09% (0.00%, 3.74%) |  |  |
|  |  |  |  |  | *Q_B_*(2) = 0.06, *p* = .971 | |  |  |
| **Kenya** | 0/69 | 0.00% (0.00%, 5.43%) | 0/120 | 0.00% (0.00%, 3.33%) | 3/143 | 0.20% (0.00%, 4.22%) |  |  |
|  |  |  |  |  | *Q_B_*(2) = 0.13, *p* = .936 | |  |  |
| **Korea** | 1/69 | 0.10% (0.00%, 6.59%) | 0/120 | 0.00% (0.00%, 3.33%) | 0/143 | 0.00% (0.00%, 2.85%) |  |  |
|  |  |  |  |  | *Q_B_*(2) = 0.04, *p* = .979 | |  |  |
| **Malawi** | 0/69 | 0.00% (0.00%, 5.43%) | 0/120 | 0.00% (0.00%, 3.33%) | 1/143 | 0.02% (0.00%, 3.28%) |  |  |
|  |  |  |  |  | *Q_B_*(2) = 0.01, *p* = .993 | |  |  |
| **Mauritius** | 0/69 | - | 0/120 | - | 0/143 | - |  |  |
|  |  |  |  |  |  | |  |  |
| **Mexico** | 0/69 | 0.00% (0.00%, 5.43%) | 2/120 | 0.13% (0.00%, 4.46%) | 0/143 | 0.00% (0.00%, 2.85%) |  |  |
|  |  |  |  |  | *Q_B_*(2) = 0.08, *p* = .961 | |  |  |
| **Netherlands** | 2/69 | 0.38% (0.00%, 7.82%) | 9/120 | 2.26% (0.00%, 9.30%) | 8/143 | 1.32% (0.00%, 6.92%) |  |  |
|  |  |  |  |  | *Q_B_*(2) = 0.34, *p* = .842 | |  |  |
| **New Zealand** | 9/69 | 6.04% (0.00%, 18.17%) | 7/120 | 1.42% (0.00%, 7.79%) | 9/143 | 1.64% (0.00%, 7.51%) |  |  |
|  |  |  |  |  | *Q_B_*(2) = 0.99, *p* = .611 | |  |  |
| **Nicaragua** | 0/69 | 0.00% (0.00%, 5.43%) | 1/120 | 0.03% (0.00%, 3.88%) | 0/143 | 0.00% (0.00%, 2.85%) |  |  |
|  |  |  |  |  | *Q_B_*(2) = 0.02, *p* = .990 | |  |  |
| **Nigeria** | 0/69 | - | 0/120 | - | 0/143 | |  |  |
|  |  |  |  |  |  | |  |  |
| **Norway** | 2/69 | 0.38% (0.00%, 7.82%) | 5/120 | 0.76% (0.00%, 6.38%) | 4/143 | 0.36% (0.00%, 4.72%) |  |  |
|  |  |  |  |  | *Q_B_*(2) = 0.05, *p* = .975 | |  |  |
| **Palestine** | 1/69 | 0.10% (0.00%, 6.59%) | 1/120 | 0.03% (0.00%, 3.88%) | 0/143 | 0.00% (0.00%, 2.85%) |  |  |
|  |  |  |  |  | *Q_B_*(2) = 0.04, *p* = .980 | |  |  |
| **Philippines** | 0/69 | 0.00% (0.00%, 5.43%) | 0/120 | 0.03% (0.00%, 3.33%) | 2/143 | 0.09% (0.00%, 3.74%) |  |  |
|  |  |  |  |  | *Q_B_*(2) = 0.06, *p* = .971 | |  |  |
| **Poland** | 0/69 | - | 0/120 | - | 0/143 | - |  |  |
|  |  |  |  |  |  | |  |  |
| **Portugal** | 1/69 | 0.10% (0.00%, 6.59%) | 0/120 | 0.00% (0.00%, 3.33%) | 0/143 | 0.00% (0.00%, 2.85%) |  |  |
|  |  |  |  |  | *Q_B_*(2) = 0.04, *p* = .979 | |  |  |
| **Puerto Rico** | 1/69 | 0.10% (0.00%, 6.59%) | 4/120 | 0.50% (0.00%, 5.71%) | 1/143 | 0.02% (0.00%, 3.28%) |  |  |
|  |  |  |  |  | *Q_B_*(2) = 0.17, *p* = .917 | |  |  |
| **Qatar** | 0/69 | 0.00% (0.00%, 5.43%) | 0/120 | 0.00% (0.00%, 3.33%) | 1/143 | 0.02% (0.00%, 3.28%) |  |  |
|  |  |  |  |  | *Q_B_*(2) = 0.01, *p* = .993 | |  |  |
| **Romania** | 2/69 | 0.38% (0.00%, 7.82%) | 6/120 | 1.07% (0.00%, 7.08%) | 3/143 | 0.20% (0.00%, 4.22%) |  |  |
|  |  |  |  |  | *Q_B_*(2) = 0.21, *p* = .901 | |  |  |
| **Russia** | 1/69 | 0.10% (0.00%, 6.59%) | 0/120 | 0.00% (0.00%, 3.33%) | 0/143 0.00% (0.00%, 2.85%) | |  |  |
|  |  |  |  |  |  | *Q_B_*(2) = 0.04, *p* = .979 |  |  |
| **Rwanda** | 0/69 | - | 0/120 | - | 0/143 | - |  |  |
|  |  |  |  |  |  | |  |  |
| **Sierra Leone** | 0/69 | 0.00% (0.00%, 5.43%) | 0/120 | 0.00% (0.00%, 3.33%) | 1/143 | 0.02% (0.00%, 3.28%) |  |  |
|  |  |  |  |  | *Q_B_*(2) = 0.01, *p* = .993 | |  |  |
| **Singapore** | 0/69 | - | 0/120 | - | 0/143 | - |  |  |
|  |  |  |  |  |  | |  |  |
| **Slovenia** | 0/69 | - | 0/120 | - | 0/143 | - |  |  |
|  |  |  |  |  |  | |  |  |
| **South Africa** | 0/69 | 0.00% (0.00%, 5.43%) | 2/120 | 0.13% (0.00%, 4.46%) | 2/143 | 0.09% (0.00%, 3.74%) |  |  |
|  |  |  |  |  | *Q_B_*(2) = 0.05, *p* = .976 | |  |  |
| **Spain** | 0/69 | 0.00% (0.00%, 5.43%) | 0/120 | 0.00% (0.00%, 3.33%) | 1/143 | 0.02% (0.00%, 3.28%) |  |  |
|  |  |  |  |  | *Q_B_*(2) = 0.01, *p* = .993 | |  |  |
| **Sri Lanka** | 1/69 | 0.10% (0.00%, 6.59%) | 1/120 | 0.03% (0.00%, 3.88%) | 0/143 | 0.00% (0.00%, 2.85%) |  |  |
|  |  |  |  |  | *Q_B_*(2) = 0.02, *p* = .980 | |  |  |
| **Sudan** | 0/69 | 0.00% (0.00%, 5.43%) | 1/120 | 0.03% (0.00%, 3.88%) | 0/143 | 0.00% (0.00%, 2.85%) |  |  |
|  |  |  |  |  | *Q_B_*(2) = 0.04, *p* = .990 | |  |  |
| **Sweden** | 4/65 | 1.41% (0.00%, 10.50%) | 3/120 | 0.29% (0.00%, 5.07%) | 21/143 | 7.40% (1.58%, 15.82%) |  |  |
|  |  |  |  |  | *Q_B_*(2) = 3.81, *p* = .149 | |  |  |
| **Switzerland** | 1/69 | 0.10% (0.00%, 6.59%) | 0/120 | 0.00% (0.00%, 3.33%) | 1/143 | 0.02% (0.00%, 3.28%) |  |  |
|  |  |  |  |  | *Q_B_*(2) = 0.03, *p* = .983 | |  |  |
| **Syria** | 0/69 | 0.00% (0.00%, 5.43%) | 0/120 | 0.00% (0.00%, 3.33%) | 1/143 | 0.02% (0.00%, 3.28%) |  |  |
|  |  |  |  |  | *Q_B_*(2) = 0.01, *p* = .993 | |  |  |
| **Taiwan** | 0/69 | - | 0/120 | - | 0/143 | - |  |  |
|  |  |  |  |  |  | |  |  |
| **Thailand** | 1/69 | 0.10% (0.00%, 6.59%) | 0/120 | 0.00% (0.00%, 3.33%) | 2/143 | 0.09% (0.00%, 3.74%) |  |  |
|  |  |  |  |  | *Q_B_*(2) = 0.06, *p* = .972 | |  |  |
| **Turkey** | 1/69 | 0.10% (0.00%, 6.59%) | 2/120 | 0.13% (0.00%, 4.46%) | 0/143 | 0.00% (0.00%, 2.85%) |  |  |
|  |  |  |  |  | *Q_B_*(2) = 0.08, *p* = .962 | |  |  |
| **Uganda** | 1/69 | 0.10% (0.00%, 6.59%) | 0/120 | 0.00% (0.00%, 3.33%) | 1/143 | 0.02% (0.00%, 3.28%) |  |  |
|  |  |  |  |  | *Q_B_*(2) = 0.03, *p* = .983 | |  |  |
| **United Kingdom** | 9/69 | 6.04% (0.00%, 18.17%) | 31/120 | 18.77% (5.43%, 30.15%) | 46/143 | 26.39% (16.31%, 37.42%) |  |  |
|  |  |  |  |  | *Q_B_*(2) = 6.30, *p* = .043 | |  |  |
| **England** | 5/69 | 2.12% (0.00%, 11.93%) | 14/120 | 4.97% (0.26%, 13.42%) | 26/143 | 10.61% (3.57%, 19.78%) |  |  |
|  |  |  |  |  | *Q_B_*(2) = 2.31, *p* = .315 | |  |  |
| **Ireland** | 0/69 | 0.00% (0.00%, 5.43%) | 1/120 | 0.03% (0.00%, 3.88%) | 3/143 | 0.20% (0.00%, 4.22%) |  |  |
|  |  |  |  |  | *Q_B_*(2) = 0.09, *p* = .958 | |  |  |
| **Isle of Wight** | 0/69 | - | 0/120 | - | 0/143 | - |  |  |
|  |  |  |  |  |  | |  |  |
| **Scotland** | 0/69 | 0.00% (0.00%, 5.43%) | 0/120 | 0.00% (0.00%, 3.28%) | 1/143 | 0.02% (0.00%, 3.28%) |  |  |
|  |  |  |  |  | *Q_B_*(2) = 0.01, *p* = .993 | |  |  |
| **Wales** | 0/69 | 0.00% (0.00%, 5.43%) | 1/120 | 0.03% (0.00%, 3.88%) | 2/143 | 0.09% (0.00%, 3.74%) |  |  |
|  |  |  |  |  | *Q_B_*(2) = 0.02, *p* = .988 | |  |  |
| **United States of America** | 13/69 | 11.26% (1.78%, 25.07%) | 17/120 | 6.96% (1.02%, 16.11%) | 19/143 | 6.24% (0.98%, 14.31%) |  |  |
|  |  |  |  |  | *Q_B_*(2) = 0.59, *p* = .757 | |  |  |
| **Zambia** | 0/69 | 0.00% (0.00%, 5.43%) | 0/120 | 0.00% (0.00%, 3.33%) | 1/143 | 0.02% (0.00%, 3.28%) |  |  |
|  |  |  |  |  | *Q_B_*(2) = 0.01, *p* = .993 | |  |  |
|  |  |  |  |  |  |  |  |  |
| **Countries of study authors** |  |  |  |  |  |  |  |  |
| **Afghanistan** | 0/361 | - | 0/522 | - | 0/487 | - |  |  |
|  |  |  |  |  |  | |  |  |
| **Australia** | 27/361 | 1.81% (0.08%, 5.23%) | 28/522 | 1.22% (0.04%, 3.67%) | 31/487 | 1.66% (0.14%, 4.45%) |  |  |
|  |  |  |  |  | *Q_B_*(2) = 0.15, *p* = .926 | |  |  |
| **Austria** | 0/361 | 0.00% (0.00%, 1.22%) | 1/522 | 0.07% (0.00%, 0.93%) | 7/487 | 0.10% (0.00%, 1.52%) |  |  |
|  |  |  |  |  | *Q_B_*(2) = 0.21, *p* = .902 | |  |  |
| **Bangladesh** | 0/361 | 0.00% (0.00%, 1.22%) | 1/522 | 0.00% (0.00%, 0.93%) | 0/487 | 0.00% (0.00%, 0.92%) |  |  |
|  |  |  |  |  | *Q_B_*(2) = 0.00, *p* = .998 | |  |  |
| **Belarus** | 1/361 | 0.00% (0.00%, 1.34%) | 0/522 | 0.00% (0.00%, 0.86%) | 0/487 | 0.00% (0.00%, 0.92%) |  |  |
|  |  |  |  |  | *Q_B_*(2) = 0.01, *p* = .996 | |  |  |
| **Belgium** | 3/361 | 0.03% (0.00%, 1.59%) | 8/522 | 0.11% (0.00%, 1.50%) | 10/487 | 0.19% (0.00%, 1.82%) |  |  |
|  |  |  |  |  | *Q_B_*(2) = 0.11, *p* = .945 | |  |  |
| **Brazil** | 0/361 | 0.00% (0.00%, 1.22%) | 1/522 | 0.00% (0.00%, 0.93%) | 7/487 | 0.07% (0.00%, 1.43%) |  |  |
|  |  |  |  |  | *Q_B_*(2) =0.12, *p* = .940 | |  |  |
| **Bulgaria** | 0/361 | 0.00% (0.00%, 1.22%) | 1/522 | 0.00% (0.00%, 0.93%) | 0/487 | 0.00% (0.00%, 0.92%) |  |  |
|  |  |  |  |  | *Q_B_*(2) = 0.00, *p* = .998 | |  |  |
| **Burkina Faso** | 0/361 | 0.00% (0.00%, 1.22%) | 0/522 | 0.00% (0.00%, 0.86%) | 1/487 | 0.00% (0.00%, 1.00%) |  |  |
|  |  |  |  |  | *Q_B_*(2) = 0.00, *p* = .998 | |  |  |
| **Burundi** | 0/361 | - | 0/522 | - | 0/487 | - |  |  |
|  |  |  |  |  |  | |  |  |
| **Canada** | 42/361 | 4.15% (1.14%, 8.46%) | 47/522 | 3.14% (0.89%, 6.38%) | 45/487 | 3.27% (0.91%, 6.66%) |  |  |
|  |  |  |  |  | *Q_B_*(2) = 0.30, *p* = .903 | |  |  |
| **Chile** | 0/361 | 0.00% (0.00%, 1.22%) | 0/522 | 0.00% (0.00%, 0.86%) | 1/487 | 0.00% (0.00%, 1.00%) |  |  |
|  |  |  |  |  | *Q_B_*(2) = 0.00, *p* = .998 | |  |  |
| **China** | 5/361 | 0.01% (0.00%, 1.46%) | 3/522 | 0.02% (0.00%, 1.08%) | 11/487 | 0.23% (0.00%, 1.92%) |  |  |
|  |  |  |  |  | *Q_B_*(2) = 0.33, *p* = .848 | |  |  |
| **Colombia** | 0/361 | 0.00% (0.00%, 1.22%) | 0/522 | 0.00% (0.00%, 0.86%) | 2/487 | 0.01% (0.00%, 1.08%) |  |  |
| **Cuba** | 1/361 | 0.00% (0.00%, 1.34%) | 0/522 | 0.00% (0.00%, 0.86%) | 0/487 | *Q_B_*(2) = 0.00, *p* = .998  0.00% (0.00%, 0.92%) |  |  |
|  |  |  |  |  | *Q_B_*(2) = 0.02, *p* = .996 | |  |  |
| **Cyprus** | 1/361 | 0.00% (0.00%, 1.22%) | 0/522 | 0.00% (0.00%, 0.86%) | 2/487 | 0.01% (0.00%, 1.08%) |  |  |
|  |  |  |  |  | *Q_B_*(2) = 0.02, *p* = .992 | |  |  |
| **Czech Republic** | 0/361 | 0.00% (0.00%, 1.22%) | 0/522 | 0.00% (0.00%, 0.86%) | 1/487 | 0.00% (0.00%, 1.00%) |  |  |
|  |  |  |  |  | *Q_B_*(2) = 0.00, *p* = .998 | |  |  |
| **Denmark** | 2/361 | 0.00% (0.00%, 1.34%) | 7/522 | 0.09% (0.00%, 1.41%) | 12/487 | 0.37% (0.00%, 2.02%) |  |  |
|  |  |  |  |  | *Q_B_*(2) = 0.37, *p* = .832 | |  |  |
| **Eritrea** | 2/361 | 0.01% (0.00%, 1.46%) | 0/522 | 0.00% (0.00%, 0.86%) | 0/487 | 0.00% (0.00%, 0.92%) |  |  |
|  |  |  |  |  | *Q_B_*(2) = 0.03, *p* = .985 | |  |  |
| **Estonia** | 0/361 | 0.00% (0.00%, 1.22%) | 0/522 | 0.00% (0.00%, 0.86%) | 2/487 | 0.01% (0.00%, 1.08%) |  |  |
|  |  |  |  |  | *Q_B_*(2) = 0.02, *p* = .990 | |  |  |
| **Ethiopia** | 0/361 | 0.00% (0.00%, 1.22%) | 1/522 | 0.00% (0.00%, 0.93%) | 0/487 | 0.00% (0.00%, 0.92%) |  |  |
|  |  |  |  |  | *Q_B_*(2) = 0.00, *p* = .998 | |  |  |
| **Finland** | 1/361 | 0.00% (0.00%, 1.34%) | 10/522 | 0.17% (0.00%, 1.68%) | 6/487 | 0.07% (0.00%, 1.43%) |  |  |
|  |  |  |  |  | *Q_B_*(2) = 0.21, *p* = .899 | |  |  |
| **France** | 4/361 | 0.06% (0.00%, 1.73%) | 4/522 | 0.03% (0.00%, 1.16%) | 8/487 | 0.13% (0.00%, 1.62%) |  |  |
|  |  |  |  |  | *Q_B_*(2) = 0.07, *p* = .965 | |  |  |
| **Germany** | 8/361 | 0.23% (0.00%, 2.30%) | 18/522 | 0.53% (0.00%, 2.49%) | 40/487 | 2.65% (0.57%, 5.84%) |  |  |
|  |  |  |  |  | *Q_B_*(2) = 3.31, *p* = .198 | |  |  |
| **Ghana** | 0/361 | 0.00% (0.00%, 1.22%) | 0/522 | 0.00% (0.00%, 0.86%) | 1/487 | 0.00% (0.00%, 1.00%) |  |  |
|  |  |  |  |  | *Q_B_*(2) = 0.00, *p* = .998 | |  |  |
| **Greece** | 3/361 | 0.03% (0.00%, 1.59%) | 1/522 | 0.00% (0.00%, 0.93%) | 2/487 | 0.01% (0.00%, 1.08%) |  |  |
| **Hong Kong** | 4/361 | 0.06% (0.00%, 1.73%) | 6/522 | 0.06% (0.00%, 1.32%) | 3/487 | *Q_B_*(2) = 0.03, *p* = .984  0.02% (0.00%, 1.26%) |  |  |
|  |  |  |  |  | *Q_B_*(2) = 0.03, *p* = .984 | |  |  |
| **Hungary** | 0/361 | 0.00% (0.00%, 1.22%) | 2/522 | 0.01% (0.00%, 1.01%) | 2/487 | 0.01% (0.00%, 1.16%) |  |  |
|  |  |  |  |  | *Q_B_*(2) = 0.03, *p* = .986 | |  |  |
| **India** | 2/361 | 0.01% (0.00%, 1.46%) | 0/522 | 0.00% (0.00%, 0.86%) | 0/487 | 0.00% (0.00%, 0.92%) |  |  |
|  |  |  |  |  | *Q_B_*(2) = 0.03, *p* = .995 | |  |  |
| **Indonesia** | 0/361 | - | 0/522 | - | 0/487 | - |  |  |
|  |  |  |  |  |  | |  |  |
| **Iran** | 1/361 | 0.00% (0.00%, 1.34%) | 0/522 | 0.00% (0.00%, 0.86%) | 0/487 | 0.00% (0.00%, 0.92%) |  |  |
|  |  |  |  |  | *Q_B_*(2) = 0.01, *p* = .996 | |  |  |
| **Israel** | 10/361 | 0.35% (0.00%, 2.62%) | 12/522 | 0.24% (0.00%, 1.87%) | 12/487 | 0.28% (0.00%, 2.02%) |  |  |
|  |  |  |  |  | *Q_B_*(2) = 0.02, *p* = .991 | |  |  |
| **Italy** | 5/361 | 0.01% (0.00%, 1.46%) | 7/522 | 0.09% (0.00%, 1.41%) | 11/487 | 0.23% (0.00%, 1.92%) |  |  |
|  |  |  |  |  | *Q_B_*(2) = 0.23, *p* = .894 | |  |  |
| **Jamaica** | 1/361 | 0.00% (0.00%, 1.34%) | 0/522 | 0.00% (0.00%, 0.86%) | 0/487 | 0.00% (0.00%, 0.92%) |  |  |
|  |  |  |  |  | *Q_B_*(2) = 0.01, *p* = .996 | |  |  |
| **Japan** | 0/361 | 0.00% (0.00%, 1.22%) | 3/522 | 0.02% (0.00%, 1.08%) | 3/487 | 0.02% (0.00%, 1.16%) |  |  |
|  |  |  |  |  | *Q_B_*(2) = 0.03, *p* = .983 | |  |  |
| **Jordan** | 0/361 | 0.00% (0.00%, 1.22%) | 0/522 | 0.00% (0.00%, 0.86%) | 3/487 | 0.02% (0.00%, 1.16%) |  |  |
|  |  |  |  |  | *Q_B_*(2) = 0.04, *p* = .980 | |  |  |
| **Kenya** | 0/361 | 0.00% (0.00%, 1.22%) | 0/522 | 0.00% (0.00%, 0.86%) | 3/487 | 0.02% (0.00%, 1.16%) |  |  |
|  |  |  |  |  | *Q_B_*(2) = 0.04, *p* = .978 | |  |  |
| **Korea** | 0/361 | 0.00% (0.00%, 1.22%) | 1/522 | 0.00% (0.00%, 0.93%) | 0/487 | 0.00% (0.00%, 0.92%) |  |  |
|  |  |  |  |  | *Q_B_*(2) = 0.01, *p* = .998 | |  |  |
| **Malawi** | 0/361 | 0.00% (0.00%, 1.22%) | 0/522 | 0.00% (0.00%, 1.22%) | 1/487 | 0.00% (0.00%, 1.00%) |  |  |
|  |  |  |  |  | *Q_B_*(2) = 0.00, *p* = .998 | |  |  |
| **Mauritius** | 0/361 | 0.00% (0.00%, 1.22%) | 0/522 | 0.00% (0.00%, 0.86%) | 1/487 | 0.00% (0.00%, 1.00%) |  |  |
|  |  |  |  |  | *Q_B_*(2) = 0.00, *p* = .998 | |  |  |
| **Mexico** | 0/361 | 0.00% (0.00%, 1.22%) | 2/522 | 0.01% (0.00%, 1.01%) | 0/487 | 0.00% (0.00%, 0.92%) |  |  |
|  |  |  |  |  | *Q_B_*(2) = 0.02, *p* = .991 | |  |  |
| **Netherlands** | 27/361 | 1.54% (0.03%, 4.82%) | 58/522 | 4.57% (1.82%, 8.18%) | 51/487 | 4.08% (1.42%, 7.71%) |  |  |
|  |  |  |  |  | *Q_B_*(2) = 2.21, *p* = .332 | |  |  |
| **New Zealand** | 15/361 | 0.76% (0.00%, 3.47%) | 10/522 | 0.17% (0.00%, 1.68%) | 9/487 | 0.16% (0.00%, 1.71%) |  |  |
|  |  |  |  |  | *Q_B_*(2) = 0.51, *p* = .775 | |  |  |
| **Nicaragua** | 0/361 | - | 0/522 | - | 0/487 | - |  |  |
|  |  |  |  |  |  | |  |  |
| **Nigeria** | 0/361 | - | 0/522 | - | 0/487 | | - |  |
|  |  |  |  |  |  | |  |  |
| **Norway** | 4/361 | 0.06% (0.00%, 1.73%) | 15/522 | 0.37% (0.00%, 2.17%) | 16/487 | 0.48% (0.00%, 2.47%) |  |  |
|  |  |  |  |  | *Q_B_*(2) = 0.39, *p* = .822 | |  |  |
| **Palestine** | 1/361 | 0.00% (0.00%, 1.34%) | 1/522 | 0.00% (0.00%, 0.93%) | 0/487 | 0.00% (0.00%, 0.92%) |  |  |
|  |  |  |  |  | *Q_B_*(2) = 0.01, *p* = .997 | |  |  |
| **Philippines** | 0/361 | 0.00% (0.00%, 1.22%) | 0/522 | 0.00% (0.00%, 0.86%) | 3/487 | 0.02% (0.00%, 1.16%) |  |  |
|  |  |  |  |  | *Q_B_*(2) = 0.04, *p* = .978 | |  |  |
| **Poland** | 0/361 | 0.00% (0.00%, 1.22%) | 1/522 | 0.00% (0.00%, 0.93%) | 2/487 | 0.01% (0.00%, 1.08%) |  |  |
|  |  |  |  |  | *Q_B_*(2) = 0.01, *p* = .994 | |  |  |
| **Portugal** | 2/361 | 0.01% (0.00%, 1.46%) | 0/522 | 0.00% (0.00%, 0.86%) | 0/487 | 0.00% (0.00%, 0.92%) |  |  |
|  |  |  |  |  | *Q_B_*(2) = 0.03, *p* = .985 | |  |  |
| **Puerto Rico** | 1/361 | 0.00% (0.00%, 1.34%) | 5/522 | 0.04% (0.00%, 1.24%) | 1/487 | 0.00% (0.00%, 1.00%) |  |  |
|  |  |  |  |  | *Q_B_*(2) = 0.06, *p* = .969 | |  |  |
| **Qatar** | 0/361 | 0.00% (0.00%, 1.22%) | 0/522 | 0.00% (0.00%, 0.86%) | 1/487 | 0.00% (0.00%, 1.00%) |  |  |
|  |  |  |  |  | *Q_B_*(2) = 0.00, *p* = .998 | |  |  |
| **Romania** | 0/361 | 0.00% (0.00%, 1.22%) | 0/522 | 0.00% (0.00%, 0.86%) | 2/487 | 0.01% (0.00%, 1.08%) |  |  |
|  |  |  |  |  | *Q_B_*(2) = 0.02, *p* = .990 | |  |  |
| **Russia** | 2/361 | 0.00% (0.00%, 1.34%) | 0/522 | 0.00% (0.00%, 0.86%) | 4/487 | 0.03% (0.00%, 1.25%) |  |  |
|  |  |  |  |  | *Q_B_*(2) = 0.06, *p* = .969 | |  |  |
| **Rwanda** | 0/361 | 0.00% (0.00%, 1.22%) | 0/522 | 0.00% (0.00%, 0.86%) | 1/487 | 0.00% (0.00%, 1.00%) |  |  |
|  |  |  |  |  | *Q_B_*(2) = 0.00, *p* = .998 | |  |  |
| **Sierra Leone** | 0/361 | - | 0/522 | - | 0/487 | - |  |  |
|  |  |  |  |  |  | |  |  |
| **Singapore** | 0/361 | 0.00% (0.00%, 1.22%) | 1/522 | 0.00% (0.00%, 0.93%) | 3/487 | 0.02% (0.00%, 1.16%) |  |  |
|  |  |  |  |  | *Q_B_*(2) = 0.03 *p* = .984 | |  |  |
| **Slovenia** | 0/361 | 0.00% (0.00%, 1.22%) | 0/522 | 0.00% (0.00%, 0.86%) | 2/487 | 0.01% (0.00%, 1.08%) |  |  |
|  |  |  |  |  | *Q_B_*(2) = 0.02, *p* = .990 | |  |  |
| **South Africa** | 4/361 | 0.00% (0.00%, 1.34%) | 1/522 | 0.00% (0.00%, 0.93%) | 5/487 | 0.05% (0.00%, 1.33%) |  |  |
|  |  |  |  |  | *Q_B_*(2) = 0.07, *p* = .964 | |  |  |
| **Spain** | 4/361 | 0.03% (0.00%, 1.59%) | 3/522 | 0.02% (0.00%, 1.08%) | 8/487 | 0.13% (0.00%, 1.62%) |  |  |
|  |  |  |  |  | *Q_B_*(2) = 0.11, *p* = .946 | |  |  |
| **Sri Lanka** | 0/361 | - | 0/522 | - | 0/487 | - |  |  |
|  |  |  |  |  |  | |  |  |
| **Sudan** | 0/361 | - | 0/522 | - | 0/487 | - |  |  |
|  |  |  |  |  |  | |  |  |
| **Sweden** | 7/361 | 0.13% (0.00%, 2.01%) | 15/522 | 0.37% (0.00%, 2.17%) | 37/487 | 2.30% (0.40%, 5.36%) |  |  |
|  |  |  |  |  | *Q_B_*(2) = 3.25, *p* = .167 | |  |  |
| **Switzerland** | 2/361 | 0.01% (0.00%, 1.46%) | 9/522 | 0.14% (0.00%, 1.59%) | 7/487 | 0.10% (0.00%, 1.52%) |  |  |
|  |  |  |  |  | *Q_B_*(2) = 0.11, *p* = .946 | |  |  |
| **Syria** | 0/361 | - | 0/522 | - | 0/487 | - |  |  |
|  |  |  |  |  |  | |  |  |
| **Taiwan** | 0/361 | 0.00% (0.00%, 1.22%) | 0/522 | 0.00% (0.00%, 0.86%) | 4/487 | 0.03% (0.00%, 1.25%) |  |  |
|  |  |  |  |  | *Q_B_*(2) = 0.08, *p* = .962 | |  |  |
| **Thailand** | 1/361 | 0.00% (0.00%, 1.34%) | 0/522 | 0.00% (0.00%, 0.86%) | 3/487 | 0.02% (0.00%, 1.16%) |  |  |
|  |  |  |  |  | *Q_B_*(2) = 0.04, *p* = .983 | |  |  |
| **Turkey** | 3/361 | 0.03% (0.00%, 1.59%) | 2/522 | 0.01% (0.00%, 1.01%) | 1/487 | 0.00% (0.00%, 1.00%) |  |  |
|  |  |  |  |  | *Q_B_*(2) = 0.03, *p* = .985 | |  |  |
| **Uganda** | 0/361 | 0.00% (0.00%, 1.22%) | 0/522 | 0.00% (0.00%, 0.86%) | 1/487 | 0.00% (0.00%, 1.00%) |  |  |
|  |  |  |  |  | *Q_B_*(2) = 0.00, *p* = .998 | |  |  |
| **United Kingdom** | 168/361 | 41.18% (34.04%, 48.44%) | 172/522 | 27.37% (21.89%, 31.10%) | 170/487 | 29.68% (23.91%, 35.68%) |  |  |
|  |  |  |  |  | *Q_B_*(2) = 9.50, *p* = .009 | |  |  |
| **England** | 161/361 | 38.94% (31.87%, 46.18%) | 162/522 | 24.98% (19.63%, 30.61%) | 155/487 | 25.80% (20.23%, 31.66%) |  |  |
|  |  |  |  |  | *Q_B_*(2) = 10.84, *p* = .004 | |  |  |
| **Ireland** | 2/361 | 0.01% (0.00%, 1.46%) | 9/522 | 0.14% (0.00%, 1.59%) | 14/487 | 0.37% (0.00%, 2.24%) |  |  |
|  |  |  |  |  | *Q_B_*(2) = 0.41, *p* = .814 | |  |  |
| **Isle of Wight** | 0/361 | 0.00% (0.00%, 1.22%) | 1/522 | 0.00% (0.00%, 0.93%) | 0/487 | 0.00% (0.00%, 0.92%) |  |  |
|  |  |  |  |  | *Q_B_*(2) = 0.00, *p* = .998 | |  |  |
| **Scotland** | 6/361 | 0.09% (0.00%, 1.86%) | 8/522 | 0.11% (0.00%, 1.50%) | 9/487 | 0.16% (0.00%, 1.71%) |  |  |
|  |  |  |  |  | *Q_B_*(2) = 0.02, *p* = .991 | |  |  |
| **Wales** | 4/361 | 0.06% (0.00%, 1.73%) | 7/522 | 0.09% (0.00%, 1.41%) | 11/487 | 0.23% (0.00%, 1.92%) |  |  |
|  |  |  |  |  | *Q_B_*(2) = 0.12, *p* = .941 | |  |  |
| **United States of America** | 115/361 | 22.94% (16.70%, 29.63%) | 243/522 | 45.31% (39.30%, 51.37%) | 270/487 | 57.08% (50.83%, 63.25%) |  |  |
|  |  |  |  |  | *Q_B_*(2) = 52.01, *p* < .001 | |  |  |
| **Zambia** | 0/361 | 0.00% (0.00%, 1.22%) | 0/522 | 0.00% (0.00%, 0.86%) | 1/487 | 0.00% (0.00%, 1.00%) |  |  |
|  |  |  |  |  | *Q_B_*(2) = 0.00, *p* = .998 | |  |  |

*Note.* Statistics for the countries of study samples, in study titles and abstracts, and of study authors within each sampling time frame. For each set of studies, each analysis yielded an overall mean point estimate (presented as percentages) with a 95% confidence interval (CI) within each sampling time frame. *k* is the number of studies included in each analysis. *n*/*k* indicates the number of countries of study samples and study authors out of the total number of studies. The *Q_B_* statistic indicated whether were significant differences in point estimates across sampling time frames. - No study samples, study titles or abstracts, or study authors for the country.

**Table S2.** PRISMA Checklist

| **Section and Topic** | **Item #** | **Checklist item** | **Location where item is reported** |
| --- | --- | --- | --- |
| **TITLE** | | |  |
| Title | 1 | Identify the report as a systematic review. | Title |
| **ABSTRACT** | | |  |
| Abstract | 2 | See the PRISMA 2020 for Abstracts checklist. | Abstract |
| **INTRODUCTION** | | |  |
| Rationale | 3 | Describe the rationale for the review in the context of existing knowledge. | Main text |
| Objectives | 4 | Provide an explicit statement of the objective(s) or question(s) the review addresses. | Appendix S1 |
| **METHODS** | | |  |
| Eligibility criteria | 5 | Specify the inclusion and exclusion criteria for the review and how studies were grouped for the syntheses. | Appendix S2 |
| Information sources | 6 | Specify all databases, registers, websites, organisations, reference lists and other sources searched or consulted to identify studies. Specify the date when each source was last searched or consulted. | Appendix S2 |
| Search strategy | 7 | Present the full search strategies for all databases, registers and websites, including any filters and limits used. | Appendix S2 |
| Selection process | 8 | Specify the methods used to decide whether a study met the inclusion criteria of the review, including how many reviewers screened each record and each report retrieved, whether they worked independently, and if applicable, details of automation tools used in the process. | Appendix S2 |
| Data collection process | 9 | Specify the methods used to collect data from reports, including how many reviewers collected data from each report, whether they worked independently, any processes for obtaining or confirming data from study investigators, and if applicable, details of automation tools used in the process. | Appendix S2 |
| Data items | 10a | List and define all outcomes for which data were sought. Specify whether all results that were compatible with each outcome domain in each study were sought (e.g. for all measures, time points, analyses), and if not, the methods used to decide which results to collect. | Appendix S2 |
|  | 10b | List and define all other variables for which data were sought (e.g. participant and intervention characteristics, funding sources). Describe any assumptions made about any missing or unclear information. | Appendix S2 |
| Study risk of bias assessment | 11 | Specify the methods used to assess risk of bias in the included studies, including details of the tool(s) used, how many reviewers assessed each study and whether they worked independently, and if applicable, details of automation tools used in the process. | n/a |
| Effect measures | 12 | Specify for each outcome the effect measure(s) (e.g. risk ratio, mean difference) used in the synthesis or presentation of results. | Appendix S3 |
| Synthesis methods | 13a | Describe the processes used to decide which studies were eligible for each synthesis (e.g. tabulating the study intervention characteristics and comparing against the planned groups for each synthesis (item #5)). | n/a |
|  | 13b | Describe any methods required to prepare the data for presentation or synthesis, such as handling of missing summary statistics, or data conversions. | n/a |
|  | 13c | Describe any methods used to tabulate or visually display results of individual studies and syntheses. | n/a |
|  | 13d | Describe any methods used to synthesize results and provide a rationale for the choice(s). If meta-analysis was performed, describe the model(s), method(s) to identify the presence and extent of statistical heterogeneity, and software package(s) used. | Appendix S3 |
|  | 13e | Describe any methods used to explore possible causes of heterogeneity among study results (e.g. subgroup analysis, meta-regression). | n/a |
|  | 13f | Describe any sensitivity analyses conducted to assess robustness of the synthesized results. | n/a |
| Reporting bias assessment | 14 | Describe any methods used to assess risk of bias due to missing results in a synthesis (arising from reporting biases). | n/a |
| Certainty assessment | 15 | Describe any methods used to assess certainty (or confidence) in the body of evidence for an outcome. | Appendix S3 |
| **RESULTS** | | |  |
| Study selection | 16a | Describe the results of the search and selection process, from the number of records identified in the search to the number of studies included in the review, ideally using a flow diagram. | Main text, Figure S1 |
|  | 16b | Cite studies that might appear to meet the inclusion criteria, but which were excluded, and explain why they were excluded. | n/a |
| Study characteristics | 17 | Cite each included study and present its characteristics. | Table S2 |
| Risk of bias in studies | 18 | Present assessments of risk of bias for each included study. | n/a |
| Results of individual studies | 19 | For all outcomes, present, for each study: (a) summary statistics for each group (where appropriate) and (b) an effect estimate and its precision (e.g. confidence/credible interval), ideally using structured tables or plots. | Table S2 |
| Results of syntheses | 20a | For each synthesis, briefly summarise the characteristics and risk of bias among contributing studies. | n/a |
|  | 20b | Present results of all statistical syntheses conducted. If meta-analysis was done, present for each the summary estimate and its precision (e.g. confidence/credible interval) and measures of statistical heterogeneity. If comparing groups, describe the direction of the effect. | Table S2 |
|  | 20c | Present results of all investigations of possible causes of heterogeneity among study results. | n/a |
|  | 20d | Present results of all sensitivity analyses conducted to assess the robustness of the synthesized results. | n/a |
| Reporting biases | 21 | Present assessments of risk of bias due to missing results (arising from reporting biases) for each synthesis assessed. | n/a |
| Certainty of evidence | 22 | Present assessments of certainty (or confidence) in the body of evidence for each outcome assessed. | Table S2 |
| **DISCUSSION** | | |  |
| Discussion | 23a | Provide a general interpretation of the results in the context of other evidence. | Main text |
|  | 23b | Discuss any limitations of the evidence included in the review. | Main text |
|  | 23c | Discuss any limitations of the review processes used. | Main text |
|  | 23d | Discuss implications of the results for practice, policy, and future research. | Main text |
| **OTHER INFORMATION** | | |  |
| Registration and protocol | 24a | Provide registration information for the review, including register name and registration number, or state that the review was not registered. | Main text, Appendix S1 |
|  | 24b | Indicate where the review protocol can be accessed, or state that a protocol was not prepared. | Main text, Appendix S1 |
|  | 24c | Describe and explain any amendments to information provided at registration or in the protocol. | Appendix S1, Footnote 1, Footnote 2 |
| Support | 25 | Describe sources of financial or non-financial support for the review, and the role of the funders or sponsors in the review. | Main text |
| Competing interests | 26 | Declare any competing interests of review authors. | Main text |
| Availability of data, code and other materials | 27 | Report which of the following are publicly available and where they can be found: template data collection forms; data extracted from included studies; data used for all analyses; analytic code; any other materials used in the review. | Main text, Appendix S1 |

Records removed *before screening:*

Duplicate records removed (n = 1,646)

Records identified from:

PsycINFO and MEDLINE (n = 3,622)

**Identification**

Records screened

(n = 1,976)

Records excluded

(n = 0)

Reports sought for retrieval

(n = 1,976)

Reports not retrieved

(n = 0)

**Screening**

Reports excluded:

Nonempirical (n = 597)

Nonhuman (n = 4)

Reports assessed for eligibility

(n = 1,976)

Studies included in review

(n = 1,375)

Reports of included studies

(n = 1,601)

**Included**

**Figure S1.** PRISMA flow diagram of the literature search. A total of 1,976 articles were published in the *Journal of Child Psychology and Psychiatry* during the three sampling time frames. A total of 601 articles were excluded because they were nonempirical or nonhuman. The 1,375 articles included in this systematic review and meta-analysis reported on 1,703 independent samples.

1. The study aim and set of analyses for study sample location in the title/abstract was preregistered and results are presented here in the supporting information file for completeness, but, based on reviewer feedback on an earlier version of this paper, this study aim is not considered in the main text.
2. A systematic search of only the PsycINFO database was preregistered. However, PsycINFO records did not include articles published from 1995 to 1998, so MEDLINE was also searched to ensure inclusion of all relevant articles.
3. Occasionally denominated as states or territories.
4. We recognize there is tremendous possible variation in cultural, lingual, historical, and socioeconomic diversity that may exist within any single country.
